# Formyl Peptide Receptor (FPR)1 Modulation by Resveratrol in an LPS-Induced Neuroinflammatory Animal Model

**DOI:** 10.3390/nu13051418

**Published:** 2021-04-23

**Authors:** Rosa Calvello, Antonia Cianciulli, Chiara Porro, Piergianni Moda, Francesco De Nuccio, Giuseppe Nicolardi, Laura Giannotti, Maria Antonietta Panaro, Dario Domenico Lofrumento

**Affiliations:** 1 Department of Biosciences, Biotechnologies and Biopharmaceutics, University of Bari, I-70125 Bari, Italy; rosa.calvello@uniba.it (R.C.); antonia.cianciulli@uniba.it (A.C.); 2 Department of Clinical and Experimental Medicine, University of Foggia, I-71100 Foggia, Italy; chiara.porro@unifg.it; 3 Nuclear Medicine Department, SS. Annunziata Hospital, I-74100 Taranto, Italy; piergiannimoda@virgilio.it; 4 Department of Biological and Environmental Sciences and Technologies, Section of Human Anatomy, University of Salento, I-73100 Lecce, Italy; francesco.denuccio@unisalento.it (F.D.N.); giuseppe.nicolardi@unisalento.it (G.N.); laura.giannotti@unisalento.it (L.G.); dario.lofrumento@unisalento.it (D.D.L.)

**Keywords:** FPR, resveratrol, nutrition, microglia, neuroinflammation

## Abstract

Among therapeutic approaches that have been investigated, targeting of receptors implicated in managing neuroinflammation has been described. One such family of receptors comprises the formyl peptide receptors (FPRs) whose ligands could play a role in host defense. The murine FPR gene family includes at least six members while in humans there are only three. The two most important members are the Fpr1 and Fpr2. Fpr1encodes murine FPR1, which is considered the murine orthologue of human FPR. Resveratrol, a non-flavonoid polyphenol rich in red wine and grapes, apart from its beneficial health effects and anti-inflammatory properties, has been reported to reduce neuroinflammation in different neurodegenerative disease models. Resveratrol anti-inflammatory responses involve the activation of the protein deacetylase sirtuin 1 (SIRT1) gene. In this work we have investigated in an LPS-based murine model of neuroinflammation the role of FPR1, examining not only if this receptor undergoes a reduction of its expression during neuroinflammation, but also whether treatment with resveratrol was able to modulate its expression leading to an amelioration of neuroinflammatory picture in a murine model of neuroinflammation. Results of this work showed that FPR1 together with SIRT1 resulted upregulated by resveratrol treatment and that this increase is associated with an amelioration of the neuroinflammatory picture, as demonstrated by the induction of IL-10 and IL1-RA expression and the downregulation of proinflammatory mediators, such as TNF-α and IL-1β. The expression and the modulation of FPR1 by resveratrol may be evaluated in order to propose a novel anti-inflammatory and pro-resolving therapeutic approach for the reduction of the detrimental effects associated with neuro-inflammation based neurodegenerative diseases and also as a promising strategy to promote human health by a diet rich in antioxidative bioactive compounds.

## 1. Introduction

Inflammation is a physiological process coordinated and controlled by the innate immune system to injury or infection caused by different types of agent in order to assure host tissue homeostasis. Neuroinflammation is defined as an inflammatory response within the brain or spinal cord and is mediated by the generation of several mediators, including cytokines, chemokines, reactive oxygen species, and secondary messengers, released by resident brain glia cells, including microglia. The invading pathogens and their components are recognized by pattern recognition receptors (PRR) including the G-protein coupled formyl peptide receptors (FPRs), which are expressed by immune cells [1]. An interesting aspect of the FPRs is their ability to recognize both proinflammatory and pro-resolving molecules, thus representing a nodal point to determine the outcome of inflammatory processes [2].

The murine FPR gene family includes at least six members while in humans there are only three. The two most important murine members are the Fpr1 and Fpr2 [3,4].

Recently, FPRs have been identified in the brain [5,6]. Microglial cells, the resident CNS macrophages, are activated after the invasion of pathogens into the brain and generatea a large number of cytokines and chemokines in order to activate other immune cells. In this respect, it has been reported that FPRs, expressed on both astrocytes and microglia, play an important role in the inflammatory responses of host defense during neurodegenerative disorders such as Alzheimer’s disease (AD) [7,8]. It has been described that bacterial LPS is able to up-regulate the function of the FPR2, that is also a specific Aβ42 receptor [9] in murine microglia cells. However, it is unclear whether an amplified microglial response to amyloidogenic peptides results in a beneficial clearance of noxious agents or in disease exacerbation by promoting inflammation. In this regard, it was described that the lack of FPRs contributes to increased mortality and altered inflammation, with the anti-inflammatory and anti-microbial responses being much more severely affected in the mouse model [10].

Astrocytes are a heterogeneous group of brain cells, characterized by types distinct in structure, distribution, and function; their activation and crosstalk with microglia is known to play a main role in local neuroinflammatory responses and brain restoration upon alteration of nervous tissue homeostasis. On the other hand, abnormal or uncontrolled activation of astrocytes is involved in the pathogenesis and progression of a large number of diseases in the central nervous system, including infection and neurodegeneration. Le et al. for the first time demonstrates that FPR and FPRL1 expressed by astrocytoma cell lines are functional, suggesting that they could be involved in the brain host defense [11].

A very recent study demonstrates that in a mouse model of AD the treatment with FPR antagonist, Boc2, significantly improved spatial memory performance, decreased neuronal damage, caused the expression of homeostatic growth factors and ameliorated microglia, but not astrocyte, reactivity. The authors found that in the hippocampus the levels of amyloid plaques were reduced by Boc2-treatment, probably through an induction of amyloid degradation [12].

Apart from the previous reports, until now, there are not many data concerning the involvement of FPRs either in protective or uncontrolled neuroinflammatory response which underlies the exacerbation of neurodegenerative diseases. Therefore, the modulation of FPR represents an intriguing new field of study for neurodegenerative diseases.

In this regard, the neuroprotective activity of medicinal plants and phytochemicals could be helpful both for prevention and therapy against neurodegenerative diseases, such as AD [13,14,15,16].

Resveratrol (trans-3,4′,5-trihydroxystilbene) is a polyphenol present in large amount in red wine, for which many pharmacologic properties, as well as cardioprotection, antioxidant and anticancer activity, are associated with its beneficial effects. It has been widely reported that this polyphenol is correlated with neuroprotective properties both in vitro and in vivo and has been also proposed to exert anti-inflammatory effects in several systems by inducing the expression of sirtuin1 (SIRT1), including in activated microglia [17,18,19].

Among therapeutic approaches that have been investigated, targeting of receptors implicated in modulating inflammation has been described. One such family of receptors comprises FPRs, whose ligands could play a role in host defense. These receptors have the ability to bind different ligands, such as formyl-Met-Leu-Phe (fMLP) and lipoxin A4 (LXA4) [20].

Therefore, the goal of this study was designed to determine the involvement of FPR1 in a mouse model of neuroinflammation. In particular, in this work we examined not only if FPR1 undergoes a change of its expression during neuroinflammation, but also if resveratrol, a natural protective compound with known anti-inflammatory effects, is able to modulate FPR1 expression leading to an amelioration of neuroinflammatory picture. 

Results obtained from this study may provide a new understanding of the function of the innate immune system during neuroinflammation, suggesting an exciting opportunity for neuroprotective effects of anti-inflammatory nutraceutical compounds, such as resveratrol, for the treatment of the inflammatory-based neurodegenerative diseases.

## 2. Materials and Method

### 2.1. Animals and Treatment Protocols

All experiments were performed following protocols approved by the Institutional Animal Committee and in accordance with European Union (EU) Directive 2010/63/EU for animal experiments. Forty-eight 129SV male mice (22–26 g, 8–10 weeks of age; Envigo, Italy) were used for experimental procedures: 24 animals received resveratrol (50 mg/kg; Sigma-Aldrich, Milan, Italy) daily by intragastric gavage for 10 days [21], while the other group of 24 mice received only the vehicle (solution of ethanol:water 1:10). On day 7 half of each group was administered either with the vehicle or LPS (*Escherichia coli*, 5 μg dissolved in sterile saline solution) by intraventricular injection [22]; mice were then sacrificed on day 10.

### 2.2. Immunohistochemistry

Immunohistochemistry procedures were carried out following the standard ABC technique (23): 10 μm thick slices were incubated with either a 1:1000 solution of mouse primary monoclonal antibody (mAb) anti-GFAP (Merck Millipore, Burlington, MA, USA), or a mouse mAb anti-Iba1 at a ratio of 1:500 (Merck Millipore) for 12 h at 4 °C, followed by incubation with a solution 1:1000 of an anti-mouse biotinylated secondary Ab (Agilent Dako, Santa Clara, CA, USA) for 1 h at room temperature. Finally, sections were incubated with extravidin peroxidase diluted 1:1500 for 1h and immunoreactions was visualized with DAB oxidation in the presence of H_2_O_2_. Sections were observed with a Nikon Eclipse E800 microscope equipped with DS-5M digital camera (Nikon Instruments S.p.A, Campi Bisenzio FI, Italy). For co-localisation experiments, sections were incubated with 1:500 anti-Iba1 mouse mAb solution (Merck Millipore) and either a 1:200 rabbit polyclonal (p)Ab anti-FPR1 or rabbit pAb anti-IL1-RA solution (Santa Cruz Biotechnology, Dallas, TX, USA) overnight. For immunocomplexes, visualization sections were incubated with goat anti-rabbit IgG Ab conjugated with Alexa fluor 546 and a Goat anti-mouse IgG Ab conjugated with Alexa fluor 488 (Thermo Fisher Scientific, Waltham, MA, USA). Sections were observed with a Zeiss LSM 800 confocal microscope (Carl Zeiss S.p.A., Oberkochen, Germany). Cell counting, body cell size and fluorescence intensity were measured with the ImageJ image processing program. Fluorescence intensity was evaluated by setting the same threshold for all the images and then calculating the percentage of pixels over the threshold on the total pixels of the image. Five sections for each experimental condition (obtained from different animals) were analyzed (at least three fields for each section).

### 2.3. Immunoblotting Assays

The striatum and hippocampus were analyzed because they are brain regions involved in the two most common neuroinflammation-based human neurodegenerative diseases, Parkinson’s Disease (PD) and AD, respectively. Hippocampus and striatum isolated after animal sacrifice were homogenized in lysis buffer (50 mM Tris pH 8, 1% Triton-X, 0.2% sodium dodecyl sulfate (SDS), 0.02 g/mL NaCl4 U/mL aprotinin, 2 μM leupeptin, 100 μM PMSF), centrifuged at 13,000× *g* and 25 μg of protein from each sample were fractionated by gel electrophoresis and transferred onto a nitrocellulose membrane. Proteins were detected by mouse MoAb anti-GFAP at a ratio of 1:1000 (Millipore), mouse MoAb anti-Iba1 at a ratio of 1:500 (Wako, Neuss, Germany), rabbit pAb anti-FPR1at a ratio of 1:200, rabbit pAb anti-IL1-RA at a ratio of 1:200; rabbit pAbanti-IL-10 at a ratio of 1:200; rabbit pAb anti-IL10-R at a ratio of 1:200; rabbit pAb anti-IL1β at a ratio of 1:250; rabbit pAb anti-TNFα at a ratio of 1:200; rabbit pAb anti-SIRT1 at a ratio of 1:500 and mouse MoAb anti-β-actin at a ratio of 1:1000 (all from Santa Cruz Biotechnology, Inc., Milan, Italy). As protein loading control in Western blotting the β-actin was used. Immunocomplexes were detected with (HRP)-conjugated secondary Abs at a ratio of 1:10000 (Bethyl, Milan, Italy) and visualized by chemiluminescence method (BioRad, Milan, Italy).

The image analysis software (Kodak Digital Science) was used to obtain densitomeric analysis of the visualized bands, employing β-actin for normalization of immunoblotting products. Results (means ± SD) were expressed as relative optical measured density.

### 2.4. Reverse Transcriptase-Polymerase Chain Reaction (RT-PCR) and Quantitative Real-Time PCR Analysis

Total RNA was extracted from all animals using Trizol Reagent according to the manufacturer’s protocol and transcribed into cDNA by reverse transcriptase using High Capacity cDNA Reverse Transcription Kit with RNase Inhibitor (Invitrogen, Milan, Italy). Then real-time PCR was carried out as previously reported [23].

The cDNA obtained was amplified in a 7300 Real-Time PCR System (Life Technologies). Briefly the target cDNA and the glyceraldehyde-3-phosphate dehydrogenase (GAPDH) reference cDNA were amplified at the same time by an oligonucleotide probe with a 5′ fluorescent reporter dye (6-FAM) and a 3′ quencher dye (NFQ). The fluorescence levels were evaluated on an ABI PRISM 7300-sequence detection system (Applied Biosystems, CA) analyzing data by the comparative threshold cycle (Ct) method. Results have been represented as fold difference.

Primer sequences for the tested genes are reported in Table 1.

### 2.5. Statistical Analysis

All statistical analyses were performed by analysis of variance (ANOVA) and Tukey’s post hoc test using Statgraphics Centurion (Statgraphics Technologies Inc., The Plains, VA, USA). Values of *p* < 0.05 were considered statistically significant.

## 3. Results

### 3.1. Astroglial Activation

**Figure 1:**
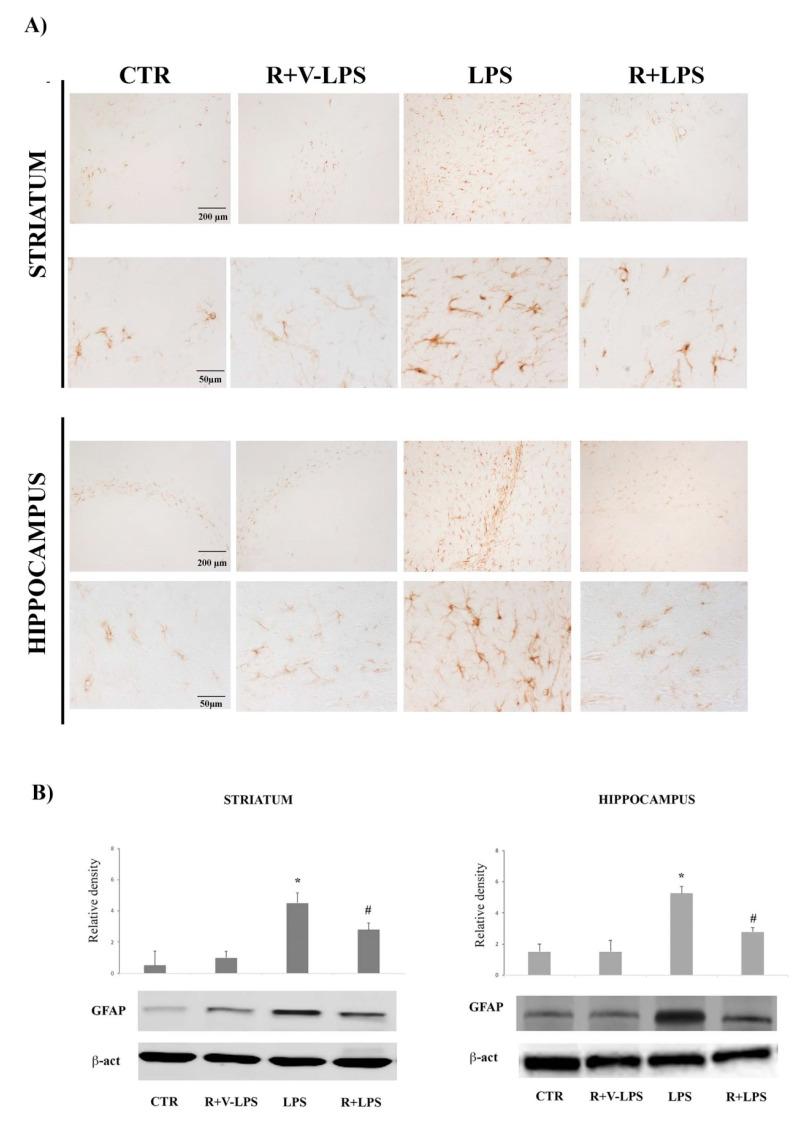
(**A**) GFAP immunoreactivity in the striatum (upper) and hippocampus (lower) in untreated controls mice (CTR), mice treated with resveratrol and LPS vehicle (R+V-LPS), LPS-treated mice (LPS) and resveratrol plus LPS treated mice (R+LPS). (**B**) Western blotting detection and densitometric analysis of GFAP expression levels in untreated controls mice (CTR), mice treated with resveratrol and LPS vehicle (R+V-LPS), LPS-treated mice (LPS) and resveratrol plus LPS treated mice (R+LPS) in the striatum (left) and hippocampus (right). For GFAP protein expression analysis values are expressed as arbitrary units after normalization against β-actin, used as a loading control. Data are represented by means ± SD (*n* = 5 in each group, 5 replicates). * *p* < 0.05 vs. control; ^#^ *p*< 0.05 vs. LPS.

Astroglial activation was evaluated by GFAP immunoreactivity, a specific marker for astrocytes, whose expression increases following nervous tissue homeostasis disruption. Images in Figure 1A report GFAP reactivity in the striatum and hippocampus of the mice control group (CTR), resveratrol and LPS vehicle (R+V-LPS)-treated mice, LPS alone (LPS) or LPS and resveratrol (R+LPS). In both CTR and R+V-LPS sections, there were no signs of reactive astrogliosis, with few immunoreactive elements mainly located around the blood vessels as part of the brain–blood barrier. LPS treatment determined a widespread increase of immunoreactivity in both striatum and hippocampus (lower magnification); in particular, immunoreactive cell bodies and ramifications were increased respect to control mice suggesting astrocyte activation (higher magnification). In R+LPS-treated mice, images show a marked decrease of the immunoreactive elements compared to the LPS ones, suggesting that in vivo administration of resveratrol reduced the presence of GFAP immunoreactive cells in both the tested brain areas. Densitometric analysis of GFAP protein expression confirmed these results showing a significant increase of GFAP protein band intensity after LPS treatment, that was significantly reduced by resveratrol (R+LPS-treated mice) treatment as reported in Figure 1B.

### 3.2. Microglia Activation

**Figure 2:**
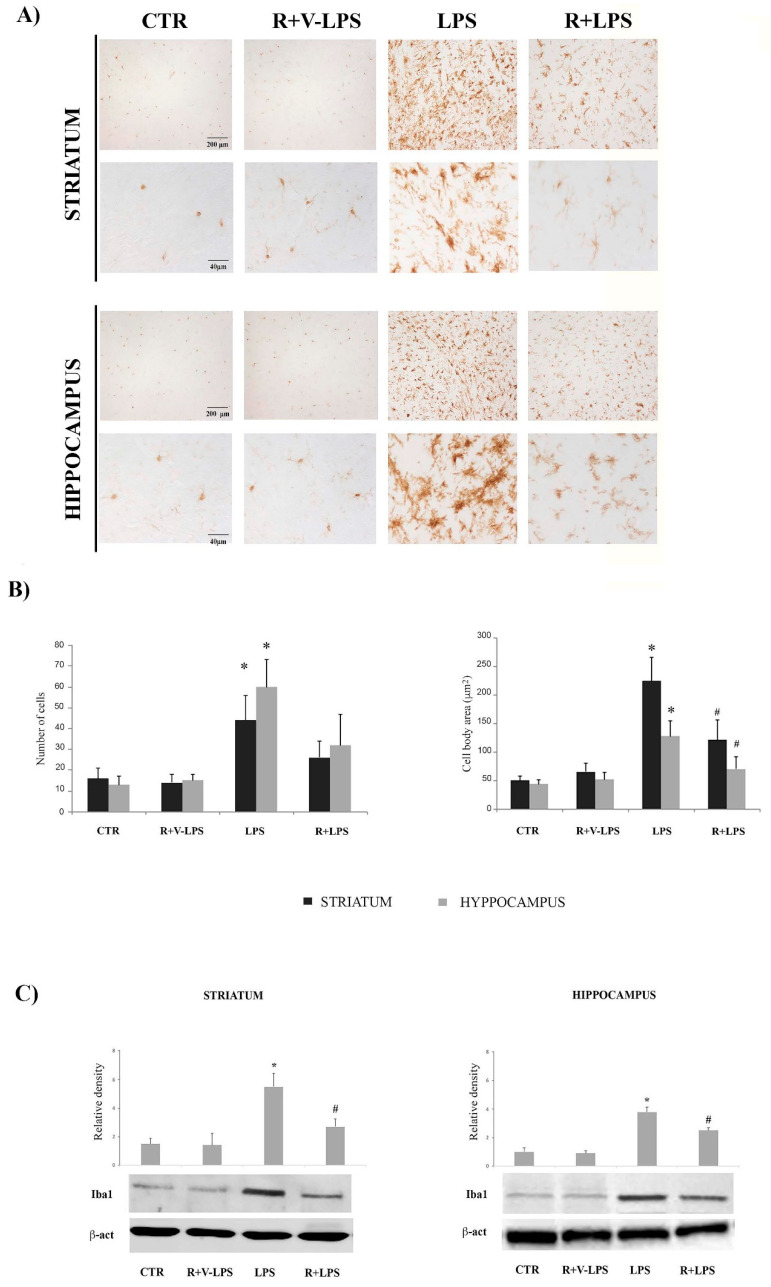
(**A**) Iba1 immunoreactive cells in the striatum (upper) and hippocampus (lower) in untreated mice (CTR), resveratrol and LPS vehicle (R+V-LPS)-treated mice, LPS-treated mice (LPS) and resveratrol plus LPS treated mice (R+LPS). (**B**) Number (per field, left) and cell body area (right) of Iba1 immunoreactive cells in untreated controls mice (CTR), resveratrol and LPS vehicle (R+V-LPS)-treated mice, LPS-treated mice (LPS) and resveratrol plus LPS treaded mice (R+LPS). Data are represented by means ± SD (*n* = 15 fields for each experimental condition, obj. 10×). (**C**) Western blotting detection and densitometric analysis of Iba1 expression levels in untreated controls mice (CTR), mice treated with resveratrol and LPS vehicle (R+V-LPS), LPS-treated mice (LPS) and resveratrol plus LPS-treated mice (R+LPS) in striatum (left) and hippocampus (right). For Iba1 protein expression analysis values are expressed as arbitrary units after normalization against β-actin, used as a loading control (*n* = 5 in each group, 5 replicates). Data are represented by means ± SD (* *p* < 0.05 vs. control; ^#^ *p* < 0.05 vs. LPS).

Iba1 (ionized calcium-binding adapter molecule-1) expression, a microglia marker, was also evaluated by immunohistochemistry assays (Figure 2A). Sections obtained from LPS-treated mice showed a more intense immunoreactivity compared to CTR and R+V-LPS ones; in particular, Iba1 positive cells were more numerous (Figure 2B, left) and showed the phenotype of activated microglia, particularly in the striatum, as demonstrated by the increased size of cell bodies (Figure 2B, right). Resveratrol administration determined the reduction of Iba1 immunoreactivity in LPS-treated mice in both striatum and hippocampus, where the immunoreactive cells showed a marked decrease of body cell size, suggesting that resveratrol was able to reduce the microglial activation associated with neuroinflammatory processes. Even in this case the data about Iba1 modulation by resveratrol were supported by results of the densitometric analysis of Iba1 protein expression, that revealed a significant increase of Iba1 protein band intensity following LPS treatment; on the other hand, it resulted significantly reduced in R+LPS-treated mice as reported (Figure 2C).

### 3.3. Resveratrol Attenuated the Pro-Inflammatory Signaling and Enhanced the Anti-Inflammatory Signaling in the Brain Specimens

**Figure 3:**
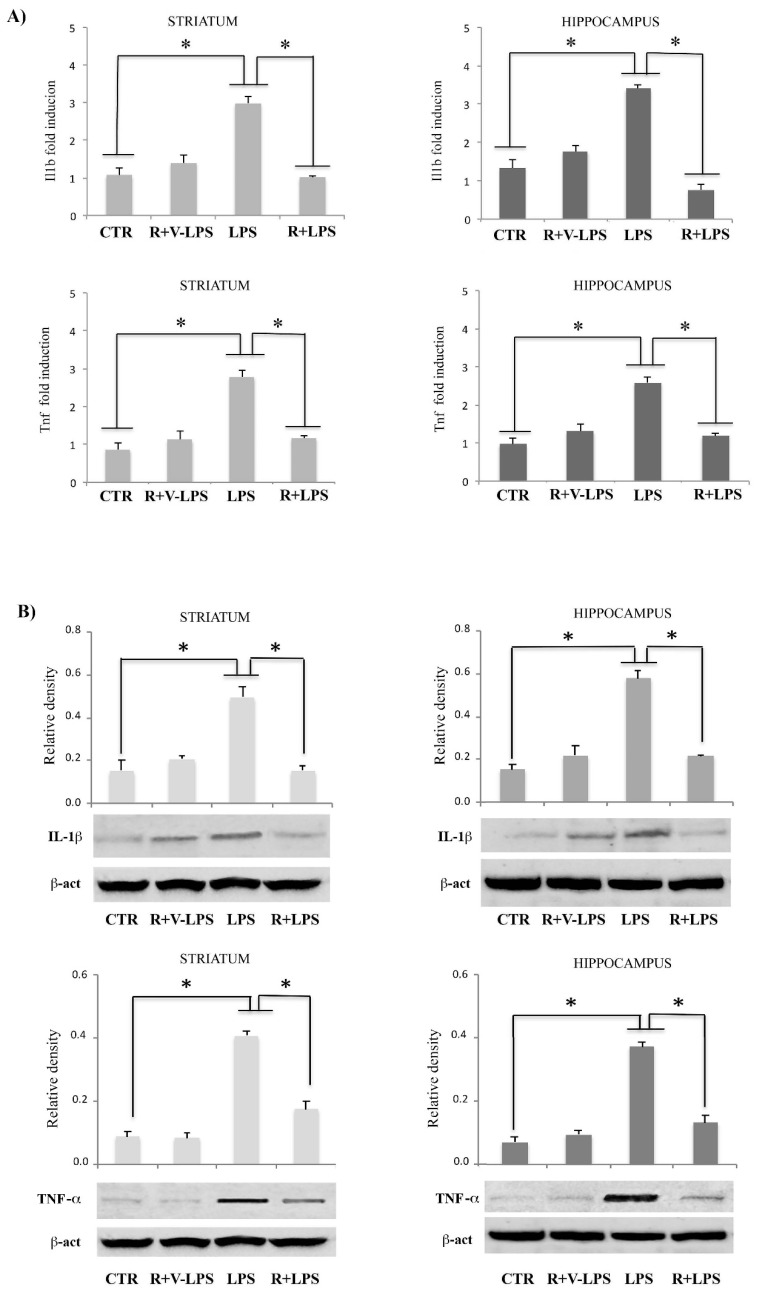
Real-time polymerase chain reaction (PCR) analysis of Il1b (upper) and Tnf (lower) mRNA expression (**A**); Western blotting detection and densitometric analysis of IL1β (upper) and TNF-α (lower) (**B**) in the striatum and hippocampus of untreated controls mice (CTR), mice treated resveratrol and LPS vehicle (R+V-LPS), LPS-treated mice (LPS) and resveratrol plus LPS-treated mice (R+LPS). For protein expression analysis values are expressed as arbitrary units after normalization against β-actin, used as a loading control. Results are shown as mRNA fold changes relative to GAPDH used as resident control. Data are represented by means ± SD (*n* = 5 per group, 5 replicates). * *p* < 0.05.

The results showed that LPS dramatically increased mRNA expression of the pro-inflammatory cytokine Il1b and Tnf in comparison to control (see Figure 3), consistent with an our previous work reporting, in a neuroinflammatory picture, an upregulation of the same genes, although tested in other brain areas [24]. Resveratrol treatment of LPS-treated mice was able to downregulate pro-inflammatory genes expression, Ilb1 and Tnf, both in striatum and hippocampus as reported in Figure 3. Similar results were observed regarding protein expressions of these mediators, confirming that resveratrol was able to regulate pro-inflammatory mediators both at gene and protein level (Figure 3).

**Figure 4:**
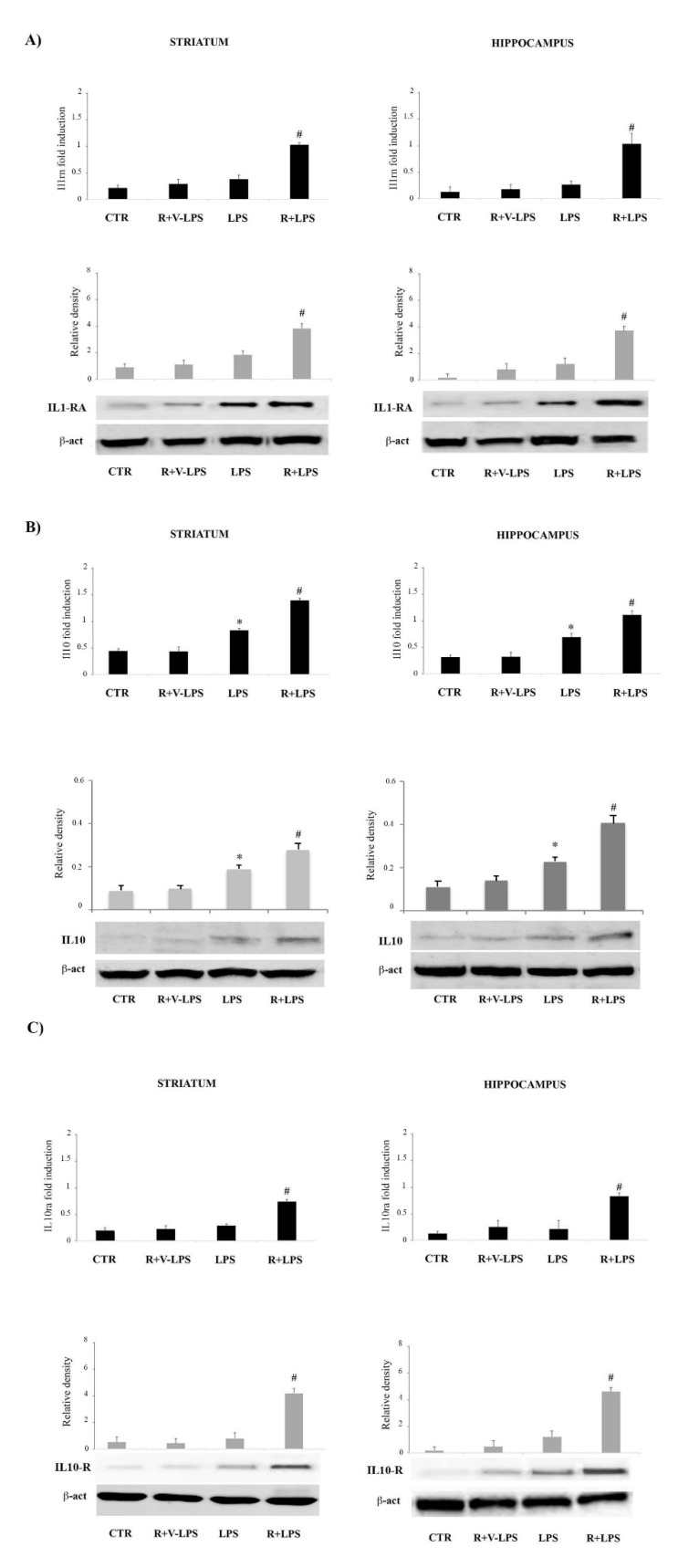
Real-time PCR analysis, Western blotting detection, densitometric analysis of (**A**) IL-1 receptor antagonist Il1rn mRNA and protein (IL1-RA) expression and (**B**) Il10 mRNA and protein (IL10) and (**C**) Il10ra mRNA and protein (IL10-R) expression in the striatum and hippocampus of untreated controls mice (CTR), mice treated with resveratrol and LPS vehicle (R+V-LPS), LPS-treated mice (LPS) and resveratrol plus LPS treated mice (R+LPS). For protein expression analysis values are expressed as arbitrary units after normalization against β-actin, used as a loading control. Results are shown as mRNA fold changes relative to glyceraldehyde-3-phosphate dehydrogenase (GAPDH) used as resident control. Data are represented by means ± SD (*n* = 5 per group, 5 replicates). * *p* < 0.05 vs. control; ^#^ *p* < 0.05 vs. LPS.

Interestingly, resveratrol resulted capable of upregulating the mRNA of anti-inflammatory genes, in terms of Il10, Il10ra and Il1rn. Consistent with the mRNA results, IL-10, IL10-R and IL1-RA protein levels resulted significantly increased by resveratrol treatment (Figure 4). Altogether these results indicate that LPS in mice brain, induces both pro- and anti-inflammatory cytokine signaling although the pro-inflammatory responses prevail, thus leading to an amelioration of the neuroinflammatory picture.

### 3.4. Formyl Peptide Receptor 1 (FPR1) and IL1-RA Immunofluorescence

**Figure 5:**
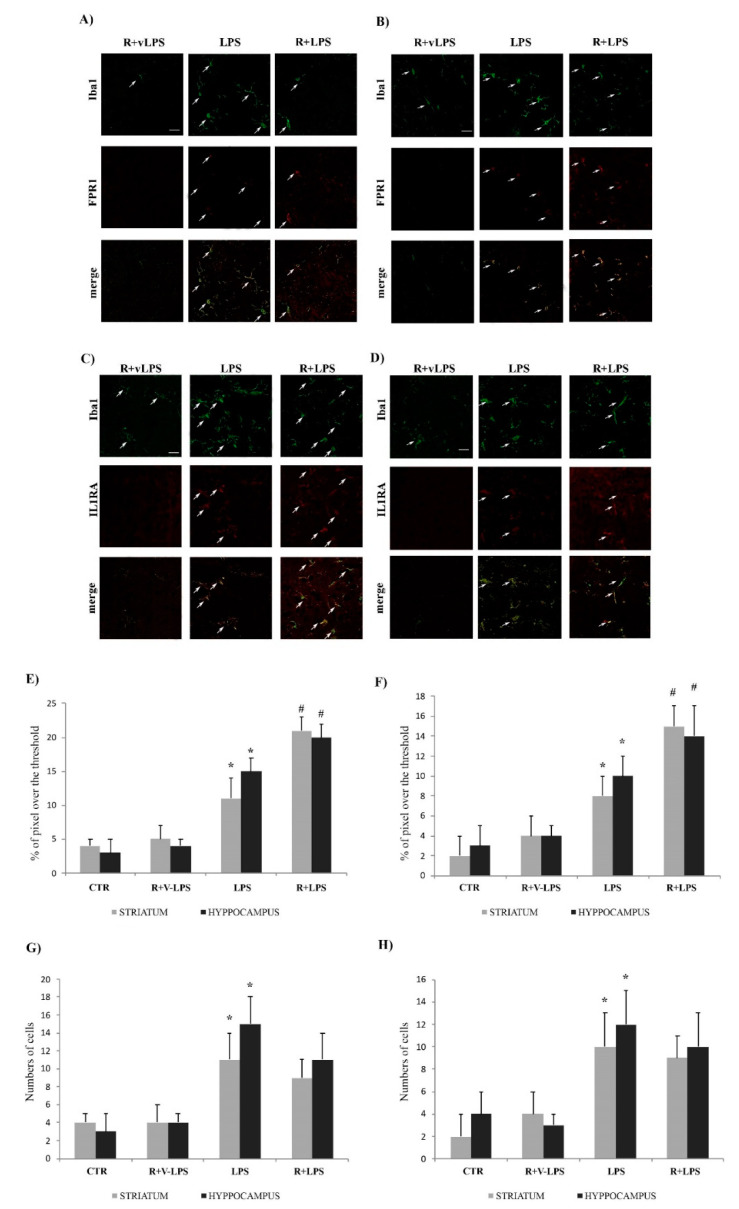
FPR1expression in Iba1-immunoreactive cells in striatum (**A**) and hippocampus (**B**). IL1-RA expression in Iba1-immunoreactive cells in striatum (**C**) and hippocampus (**D**); arrows show immunoreactive cells; scale bar: 20 μm. Fluorescence intensity of FPR1 (**E**) and IL1-RA (**F**) immunoreactive cells in striatum and hippocampus evaluated by setting the same threshold for all the images and then calculating the percentage of pixels over the threshold on the total pixels of the image. Number of FPR1 (**G**) and IL1-RA (**H**) immunoreactive cells per field; *n* = 15 for each experimental condition (obj. 40×); CTR: untreated controls mice; R+V-LPS: mice treated with resveratrol and LPS vehicle; LPS: mice treated with LPS; R+LPS: resveratrol plus LPS treated mice; data are represented by means ± SD (* *p* < 0.05 vs. control; ^#^ *p* < 0.05 vs. LPS).

To evaluate the potential involvement of FPR1 and IL1-RA in the immunomodulatory effects exerted by resveratrol on microglia in LPS-treated animals, immunofluorescence double-staining assays were carried out (Figure 5). In CTR (not shown) and R+vLPS the expression of FPR1 was very low in both striatum and hippocampus (Figure 5A,B). The inflammatory stimulus induced its expression (LPS); anyway, sections from animals treated with both LPS and resveratrol (R+LPS) showed a more intense FPR1 immunoreactivity (Figure 5E). IL1-RA immunofluorescence staining showed similar results (Figure 5C,D): the expression of this protein augmented in microglia of LPS-treated animals with respect to untreated ones (CTR and R+V-LPS); resveratrol administration in LPS-activated microglia induced further expression of IL1-RA in both striatum and hippocampus (Figure 5F). Immunoreactive cell counting confirmed that both LPS and R+LPS treatments induced the expression of FPR1 and Iba1; however, no significant difference in cell numbers was evidenced between these experimental conditions (Figure 5G,H). These results suggest that the resveratrol anti-inflammatory action could involve, at least in part, the induction of the FPR1 and IL1-RA expression.

### 3.5. mFPR1 and Sirtuin 1 (SIRT1) Expression

**Figure 6:**
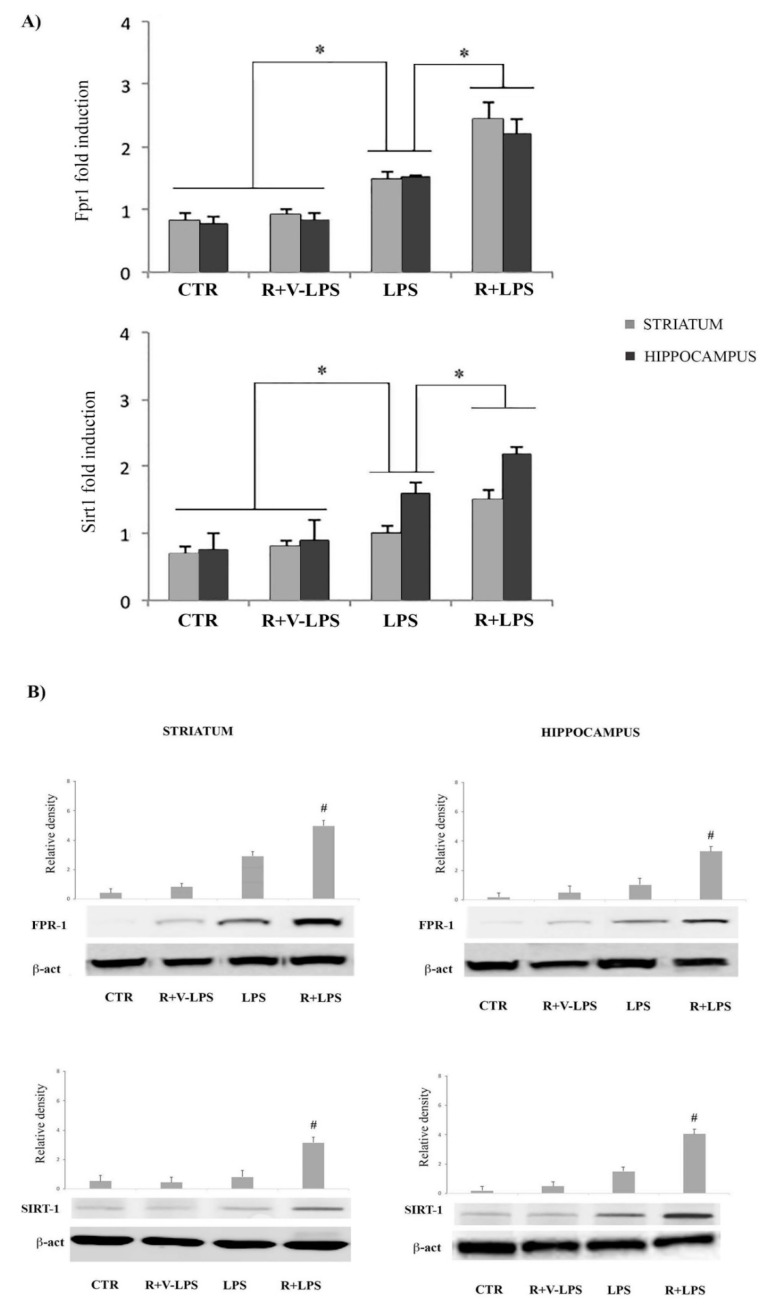
(**A**) Real-time PCR analysis of Fpr1 (upper) and Sirt1 (lower) mRNA expression in the striatum and hippocampus of untreated controls mice (CTR), mice treated with resveratrol and LPS vehicle (R+V-LPS), LPS-treated mice (LPS) and resveratrol plus LPS treated mice (R+LPS). (**B**) Western blotting detection and densitometric analysis of FPR1 (upper) and SIRT1 (lower). Real-time PCR analysis results represent the mRNA fold changes relative to GAPDH used as resident control. Protein expression analysis values are expressed as arbitrary units after normalization against β-actin, used as a loading control. All values are expressed as means ± SD (*n* = 5 per group, 5 replicates). * *p* < 0.05 significantly different; ^#^ *p* < 0.05 vs. LPS.

In this study we examined the expression of mFPR1and SIRT1 both isolated from animals, both in terms of mRNA and protein expression. As shown in Figure 6 the mFPR1 mRNA expression was evident in the striatum and hippocampus, both in control and in treated animals. In this context, we noticed that the transcripts for this receptor resulted downregulated in treated animals compared to controls. Interestingly, in LPS-treated mice that received resveratrol the mRNA levels resulted significantly increased in comparison to LPS-treated mice in absence of resveratrol. In this respect, we observed that resveratrol treatment determined a significantly increase of mFPR1 expression in comparison to untreated mice (control).

Concerning mRNA SIRT1 analysis, we observed that resveratrol treatment determined a significant increase of its expression in LPS-treated mice receiving resveratrol in comparison to LPS-treated mice, thus confirming a prevalence of the anti-inflammatory responses in resveratrol-treated mice (Figure 6).

## 4. Discussion

Although there is no specific cure for neuroinflammation-based neurodegenerative diseases, some treatments may markedly improve the pathology symptoms. For example, different studies indicate that nutrition may play an important role in neurodegenerative diseases, such as PD or AD. Indeed, many active ingredients present in certain food groups have been identified as promising in eliciting neuroprotection [25,26,27].

Many searches, including clinical reports, have described the protective effects of resveratrol in different pathological conditions. In this regard, evidence showed that resveratrol plays beneficial effects on cancer, respiratory, metabolic, cardiovascular and neurodegenerative diseases by modulating the inflammatory responses [28]. The protective benefits of resveratrol are attributable, at least in part, to its action in a variety of intracellular signalling pathways, including the regulation of the HO-1/MAPK, PI3K/Akt, NF-kB and SIRT1 expression [29,30,31,32].

Resveratrol was reported to reduce TNF-α and NO production in LPS induced primary microglia to prevent the activation of microglial BV-2 cells induced by LPS [33], to inhibit in rat primary microglia the production of PGE2 and free radicals [34], and to modulate inflammatory responses in microglia and astrocyte [35].

Additionally, it was described how resveratrol inhibits both in vitro and in vivo the migration, adhesion and invasion of glioblastoma-initiating cells, through the suppression of the PI3K/Akt/NF-κB cascade [36].

An innovative therapeutic strategy for treating neurodegenerative disease is represented by the attenuation of neuroinflammation, and several in vivo studies showed that resveratrol effectively reduces the increased expression of pro-inflammatory cytokines, inhibiting NF-κB and reducing both p38-MAPK and JNK phosphorylation, decreases COX-2 and iNOS expression and inhibits microglial and astroglial activation [37,38,39].

All these observations suggest that the suppression of inflammation is associated with the neuroprotective effects of resveratrol.

In addition to Toll-like receptors and nucleotide-binding oligomerization domain receptors, FPRs belong to the PRRs based on their capacity to recognize both bacteria and host-derived agonists [40]. In this work we aimed to investigate in a murine model of neuroinflammation the expression and the possible modulation of FPR1 by in vivo resveratrol treatment.

Results of this study demonstrated that FPR1 is positively modulated by resveratrol treatment in the striatum and hippocampus in a similar manner, and that this upregulation is detectable in a picture of neuroinflammation reduction, thus suggesting a possible protective action for FPR1 in neurodegenerative diseases like PD and AD, in which the inflammation role in both pathogenesis and progression have been established.

It is well known that FPR1 is mainly expressed on defense cells with chemotactic or phagocytic activity, including neutrophils, dendritic cells, monocytes, macrophages and glia cells, although it has been detected also in non-phagocytic cells, such as neurons [41,42]. Our results show a significant reduction of FPR1 expression in an LPS-treated mouse in comparison to control animals, whereas resveratrol was able to upregulate the FPR1 expression, accompanied by a significant reduction of typical signs of neuroinflammation. The contribution of the FPRs during inflammatory response is still under debate. Whereas a study showed a reduction of the inflammatory response in FPR-deficient mice, another one showed an increase of the inflammation, thus strengthening the conclusion for an anti-inflammatory role of the FPRs [43,44].

Studies on mouse models of bacterial meningitis revealed a stronger glial cell marker expression in FPR1-deficient mice and an increase of activated microglial cells and neutrophil infiltration in FPR1- or FPR2-deficient mice, thus suggesting that FPRs exert an important role in the immune responses within the CNS and that the lack of these receptors leads to a dysregulation of the inflammatory response compared with wild-type mice. In this context, these researchers have observed that the proinflammatory reactions in FPR1 and FPR2 deficient cells resulted significantly increased [10,45]. Moreover, it has been demonstrated that FPRs play a major role in crotoxin anti-inflammatory activities that are inhibited by the pretreatment with Boc2, a selective antagonist of FPRs [46,47].

Together with its role in the modulation of detrimental pro-inflammatory responses, FPR1 is involved in mechanisms promoting neurogenesis. Fusco et al. reported that Fpr1 promoted the differentiation of neuronal stem cells into neurons and reduced their differentiation into astrocytes via the PI3K/Akt pathway [48].

In this respect we have examined how anti-inflammatory treatment, represented by in vivo resveratrol administration, is able to modulate FPR1 expression leading to an amelioration and a resolution of a neuroinflammatory picture. It is well known that FPR1 binds several ligands, such as the mitochondrial and pro-inflammatory bacterial N-formylpeptides, as well as the anti-inflammatory agonists lipoxin A4 and annexin-1 [40]. Previous investigators have reported a potential anti-inflammatory role for FPR1 being a receptor for annexin-1, which mediates anti-inflammatory effects [49].

Moreover, it has been reported that resveratrol is able to augment production of lipoxin A4 playing beneficial effects [50]. It is possible that up-regulation by resveratrol treatment of the endogenous ligand, such as lipoxin 4, may be responsible for a positive regulation of FPR1 that, in turn, could modulate anti-inflammatory responses. However, this hypothesis, although suggestive, must be further verified in an in vivo or in vitro experimental system.

Another important point of discussion is the modulation of IL1-RA. In fact, to characterize more deeply the innate immune response in this in vivo model of neuroinflammation the IL-1 receptor antagonist expression was also determined. IL1-RA normally, inhibits the pro-inflammatory response of IL-1β. While IL1-RA resulted similarly expressed in both control animal and LPS-treated mouse, it resulted upregulated in LPS-treated mice after resveratrol administration. Along with the variation in pro-inflammatory cytokine expression, the upregulation of FPR1 and IL1-RA resulted in a significant increase of anti-inflammatory mediators in resveratrol treated mice. In this respect it is worth mentioning that the lack of FPRs is accompanied by a strong decrease of anti-inflammatory response including IL-1RA expression in vivo and in vitro as previously reported [10].

In our previous experiments we showed that in a mouse model of PD-like disease, administration of resveratrol was able to reduce dopaminergic cell loss by modulating pro-inflammatory responses [21]. The results of the present work evidence an upregulation of SIRT1 in animals that received resveratrol in combination with LPS. In this context, sirtuins are the class III of histone deacetylases and are nicotinamide adenine dinucleotide (NAD+)-dependent enzymes. Among seven sirtuins, SIRT1 exerts a critical role in modulating a wide range of physiological processes, including inflammatory responses [51]. Numerous studies indicate the protective effects of SIRT1 in neuroinflammation-related diseases and resveratrol represents a potent activator of SIRT1. In this regard, it was reported that resveratrol is able to inhibit the expression of inflammatory factors in LPS-induced activation of microglia cells by upregulation of SIRT1 [52] thus representing an optimal marker to evaluate the anti-inflammatory response. In this respect, our results are in line with previous observations suggesting the ability of resveratrol to repress inflammation process through promoting the expression of SIRT1 [53].

## 5. Conclusions

To the best of our knowledge, our results were not only unexpected but also unprecedented, since they reveal a role for the formyl peptide receptors in the immune response modulation in an in vivo mouse model of neuroinflammation. In particular, the modulation exerted by resveratrol on FPR1 expression leads to an increase of anti-inflammatory responses, as demonstrated by the downregulation of proinflammatory mediators, such as TNF-α and IL-1β and the induction of the expression of IL-1RA and IL-10. In particular, IL-10 may be involved in the upregulation of FPR1 since previous observations reported a modulation of formyl peptide receptor expression by IL-10 in human monocytes and neutrophils [54].

**Figure 7:**
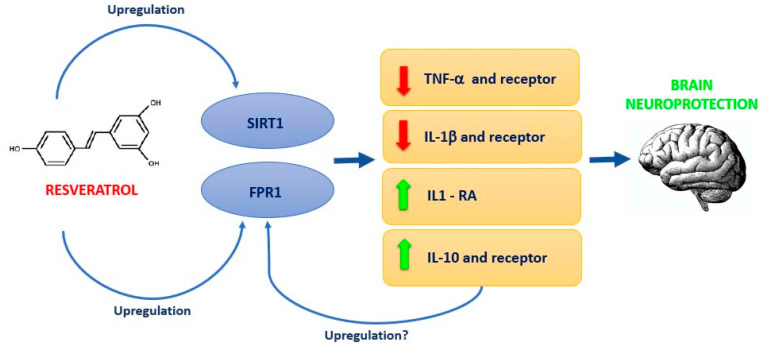
Schematic presentation of the possible mechanism for resveratrol neuroprotection in a mouse model of LPS-induced neuroinflammation. Resveratrol induces FPR1 and SIRT1 upregulation that could improve neuroinflammatory condition and IL-10 may be involved in the upregulation of FPR1.

These observations suggest that resveratrol administration may represent a novel anti-inflammatory and pro-resolving therapeutic approach for the reduction of the detrimental effects associated with neuroinflammatory processes (see Figure 7). Further studies are now needed to investigate the downstream effectors of the FPR pathway.

## Figures and Tables

**Table 1:** Primer sequences for the tested genes.

cDNA Target	Gene Official Name	Sequence (5′->3′)	Sequence References
IL-1β	*Il1b*	Fw 5′-GCAGCAGCACATCAACAAGAGC-3′ Rw 5′-GTCCTCATCCTGGAAGGTCCACG-3′	NM_008361.2
IL-1RI	*Il1r1*	Fw 5′-TGCAAAGTGTTTCTGGGAAC-3′ Rw 5′-ATATTGCCCCCACAACCAAG-3′	NM_008362.2
IL1-RA	*Il1rn*	Fw 5′-TGCAAATGAGGGAGTCTGGT-3′ Rw 5′-GCAATGAATTCTAGGCTCAGGC-3′	XM_006497727.3
TNF-α	*Tnf*	Fw 5′-GGCAGGTCTACTTTGGAGTCATTGC-3′ Rw 5′-ACATTCGAGGCTCCAGTGAATTCGG-3′	NM_013693.2
IL-10	*Il10*	Fw 5′-TAACTGCACCCACTTCCCAG-3′ Rw 5′-AGGCTTGGCAACCCAAGTAA-3′	NM_010548.2
IL10-R	*Il10ra*	Fw 5′-TCTTCAGTTCTCAGGACGCC-3′ Rw 5′-GCAATGAATTCTAGGCTCAGGC-3′	NM_001324486.1
FPR1	*Fpr1*	Fw 5′-ATTGCACTGGACCGCTGTAT-3′ Rw 5′-CCAGGGGGAGAAGTCGAAAG-3′	NM_013521.2
SIRT1	*Sirt1*	Fw 5′-CGTCTTATCCTCTAGTTCTTGTG-3′ Rw 5′-ATCTCCATCAGTCCCAAATCC-3′	NM_019812
GAPDH	*Gapdh*	Fw 5′-ACCACAGTCCATGCCATCAC-3′ Rw 5′-TCCACCACCCTGTTGCTGTA-3′	BC_085315.1

## Data Availability

All data presented in this study are available on request from the corresponding author. The data are not uploaded in publicly accessible databases.
